# The impact of pharmacists’ interventions within the closed loop immunosuppressant management process on kidney transplant recipients: a retrospective cohort study

**DOI:** 10.3389/fimmu.2025.1553786

**Published:** 2025-09-08

**Authors:** Hongxia Chen, Chuan Li, Shengsong Ou, Xiaoyu Chen

**Affiliations:** ^1^ Department of Clinical Pharmacy, People’s Hospital of Guangxi Zhuang Autonomous Region, Nanning, Guangxi, China; ^2^ Department of Transplantation, People’s Hospital of Guangxi Zhuang Autonomous Region, Nanning, Guangxi, China

**Keywords:** immunosuppression therapy, immunosuppressant management, transplantation, pharmacists, clinical practice

## Abstract

**Introduction:**

Nowadays, kidney transplant recipients’ primary challenge is improving graft function. However, they are rarely provided effective long-term instructions on immunosuppressant use after transplant. This study aimed to describe the experiences of a pharmacist-led, closed-loop immunosuppressant service (PLIS) in the transplant center of a general hospital in China.

**Methods:**

A retrospective pre-and post‐intervention study was conducted in the transplantation department in a general hospital. Of the 347 patients receiving kidney transplants from August 2022 to August 2024 were enrolled. Eligible subjects were assigned into two groups (pre‐intervention group and post‐intervention group) according to the date (1 August 2023) when the pharmacist commenced participation in the post‐transplant management for kidney transplant recipients. The intra-patient variation in immunosuppressant trough concentrations (C_min_) before and after the intervention was defined as the primary outcome. The secondary outcome was to assess the impact on renal function.

**Results:**

Among 347 patients (August 2022–2024), those managed post-intervention (from August 2023) showed improved target trough concentration (C_min_) attainment versus pre-intervention: tacrolimus (TAC, 72.4% vs. 58.3%, *P*=0.012), cyclosporine (CsA, 63.7% vs. 46.5%, *P*=0.037), mycophenolate (MMF, 76.0% vs. 65.3%, *P*=0.025), and sirolimus (SRL, 80.2% vs. 51.9%, *P*=0.018). Compared to pre-intervention, the percentage coefficient of variation (%CV) decreased significantly for TAC (18.28% vs. 8.92%, *P*=0.031) and CsA (22.97% vs. 7.14%, *P*=0.004) post-intervention, while MMF maintained high variability (CV >30%). SRL variability declined at 6–12 months (17.02% vs. 26.05%, *P*=0.194). Renal function improved post-intervention, with reductions in serum creatinine, urea nitrogen, cystatin C, and microproteinuria (*P*<0.05).

**Conclusion:**

PLIS enhanced immunosuppressant management precision and graft outcomes, demonstrating its utility in standardizing post-transplant care.

## 1 Introduction

Advances in maintenance immunosuppression have improved solid organ transplantation outcomes dramatically over the past three decades. Rejection minimization and allograft survival depend heavily on uninterrupted immunosuppressive therapy. However, immunosuppressant-related issues are common among kidney transplant recipients. Agents used vary based on center-specific protocol, physicians’ expertise, ability to cover copays, types of transplanted organs and recipient characteristics, and acceptability. Besides, the continued management of comorbidities, a substantial new medication regimen (on average eight drugs per day), and intensive follow-up care constitute a significant challenge for these patients (1). After transplantation, the medication discrepancies can lead to fluctuating immunosuppression levels, which in turn may result in transplant rejection, infection, and/or adverse drug reaction (2). In addition, low adherence to immunosuppressive therapy is common among adult kidney recipients and is a major cause of transplant failure, especially in the first year after transplantation (3). Previous research data show that 58.7% of transplant rejections were associated with non-adherence, highlighting its importance in proper treatment management (4). The rate of non-adherence to immunosuppressants ranges from 36% to as much as 80% in kidney transplant recipients in particular–indicating a specific risk of rejection in this population (5). Medication adherence reportedly falls sharply at 9 months post-transplantation, which emphasizes the importance of long-term follow-up (6, 7). Going further, a finding points to the need for multilevel interventions beyond the patient level, targeting transplant center practice patterns as an approach to tackle nonadherence (8, 9).

Given the positive impact of appropriate medication management on graft outcomes and therefore of patient survival and graft function, the pharmacist’s role in the kidney transplantation team has evolved over recent decades. Studies demonstrate that pharmacists play a comprehensive role in the management of kidney transplant recipients in the inpatient setting, including pre-transplant care and readiness for transplantation, with evidence of enhanced clinical outcomes (10, 11). Furthermore, a report has shown that a collaborative pharmacy practice agreement was developed between physicians and pharmacists and implemented into a renal transplant clinic, and pharmacists have the advantage of reducing physician and nurse workload related to prescribing by utilizing their expertise to take over certain tasks (12). A health economics study demonstrated that a mHealth-enabled, pharmacist-led intervention significantly reduced hospitalization costs for payers over 12 months and has a positive return on investment (13).

At our institution, several patients experienced delayed graft function (DGF) and prolonged hospitalization for inappropriate use of immunosuppressants due to a lack of consensus recommendations. With the expansion of transplantation services and integration of an inpatient transplant clinical pharmacist, transplant specialists at our hospital requested standardizing the process to improve patient care and safety outcomes. Thus, an institution-specific pharmacist-driven protocol for standardizing the management of immunosuppression in adult kidney transplant recipients was established. The primary objective of this initiative was to evaluate the effect of protocol implementation on patients’ therapeutic adherence, the proportion of immunosuppression variability, and graft function; the secondary objective was to develop an approach that could improve patients’ long-term medication adherence.

## 2 Methods

### 2.1 Study design and setting

This was a retrospective cohort study conducted at the transplant center in a general hospital in Guangxi, China. The study was approved by the Ethics Committee of the People’s Hospital of Guangxi Zhuang Autonomous Region (No. KY-11T-2024-202). The patient gave written informed consent for the use of his medical data, which were sampled and stored by privacy regulations. Between August 2022 and August 2024, 481 patients underwent living-donor or deceased-donor kidney transplantation. Exclusion criteria: (1) Children recipients (<18 year). (2) Multiorgan transplant recipients or previous transplant history (3) Recipients of non-standard immunosuppression regimens, CYP3A4 modifiers, or extended-release formulations. (4) Impaired self-administration or communication capacity. (5) Active viral infections within 180 days post-transplant. (6) ICU admission or acute kidney injury within 48 hours post-transplant. 347 patients were enrolled and assigned into two groups (162 in pre‐intervention arm and 185 in post‐intervention arm) based on the date when the pharmacist commenced participation in the post‐transplant management for kidney transplant recipients. Service and surgical protocols did not change during the study period. The follow-up time was 12 months post-transplantation for each patient.

Induction therapy with monoclonal or polyclonal agents was used according to the condition of the patient. TAC, MMF, and glucocorticoids (GC) are standard initial immunosuppressive strategies to prevent allograft rejection in our center. A small number of patients are treated with CsA regimens due to TAC adverse reactions (such as hyperglycemia or neurotoxicity). Patients with intractable diarrhea were switched to SRL, GC, and TAC regiments three months after surgery.

### 2.2 Pre-intervention and post-intervention phase

**Figure 1:**
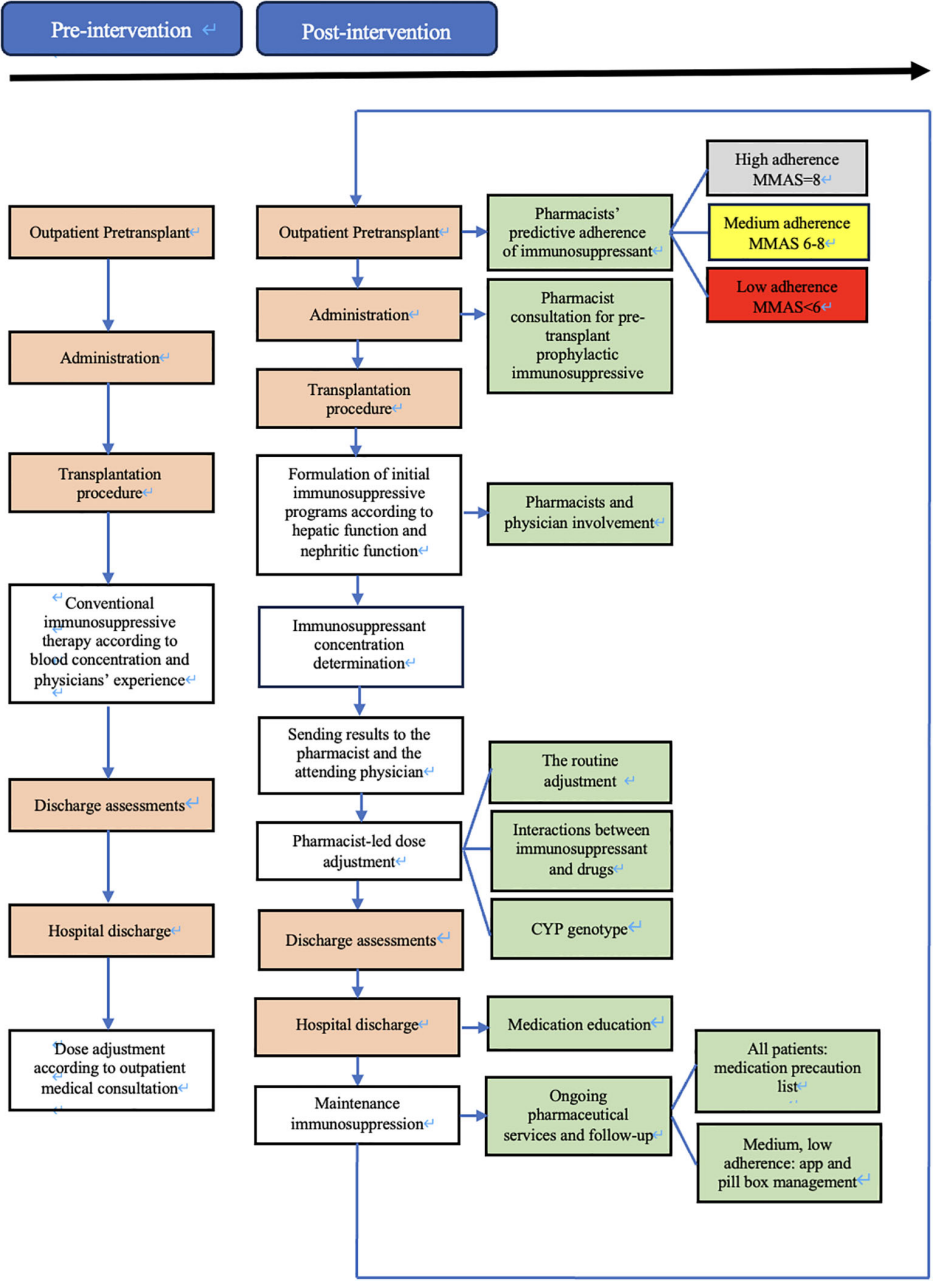
Flowchart of study patients.

During the pre-intervention phase, patients included will be managed according to usual care. If educational and/or clinical pharmacy activities already exist in some clusters, they will be maintained with the collection of this information (**Figure 1**).

On 1 August 2023, immunology pharmacists joined the transplantation team to support immunosuppressant management for kidney transplant recipients. The intervention protocol was created and implemented by the immunology pharmacist along with transplant physicians. The protocol required the immunology pharmacists to evaluate candidates’ medication adherence, manage the dose of immunosuppressant, provide medication education, and provide ongoing personalized pharmaceutical services and follow-up (**Figure 1**). To standardize this intervention over the various pharmacists, they have received a one-year training in medication reconciliation procedures, pharmaceutical interviews and consultations, and therapeutic follow-up by an experienced clinical pharmacist who is accredited by the China Hospital Association Pharmaceutical Affairs Committee.

During the pre-transplantation phase, outpatient transplant candidates were provided with the eight-item medication adherence visual analog scale (MA-VAS) for adherence-specific evaluation. The total score on the MA-VAS can range from 0 to 8, where higher scores indicate higher adherence. The degree of adherence was determined according to the score resulting from the sum of all the correct answers: low adherence (< 6 points), medium adherence (6 to < 8 points), and high adherence (8 points). Evaluation documents (candidates’ information, MA-VAS sheet, pharmaceutical consultations, etc.) will be sent to the attending physician.

At the time of post-transplantation, the assigned pharmacist comprehensively evaluated the immune status of the patient by the clinical symptoms, and laboratory tests, and provided the initial dose suggestion (e.g., immunosuppressant type, dose, and frequency) based on the patient’s conditions (e.g., liver and kidney function, underlying disease, etc.), and the best clinical evidence (as informed by evidence-based practice guidelines) (**Table 1**). Furthermore, the implementation of all therapeutic regimens is contingent upon comprehensive understanding and agreement by both patients and their family members. Finally, the immunosuppressive therapeutic regimen for individual requires collaborative approval by the medical teams. The accepted initial dose was first adjusted according to the target drug concentrations. All new drug concentration results were automatically e‐mailed to an assigned pharmacist and the attending physician in real-time. Automatic e‐mails were generated via PASS Pharm Care^®^ (Medicom, Inc., Sichuan, China), a clinical decision support platform used for clinical pharmacy service activities at our institution. If the concentration was not within the target range, the pharmacist then adjusted the dose based on the routine adjustment protocol, cytochrome P450 (CYP) genotype (14), and drug interactions (**Table 2**). Once a patient has achieved a therapeutic steady state with troughs within the goal range, the frequency of monitoring is decreased to two or three times weekly at the pharmacist’s discretion. A report of each adjustment was placed in the patient’s file and was available to the medical team.

**Table 1:** Formulation of initial dose according to hepatic and renal function.

Drug	Mild-moderate impairment (Child-Pugh A or B)	Severe impairment (Child-Pugh C)	Recommended monitoring
Cyclosporine A	5-7.5mg/kg bid	5-7.5mg/kg bid	Continue present management
Tacrolimus	0.075-0.15mg/kg bid	0.075 mg/kg bid or lower	Monitor level once or twice weekly
Mycophenolate	0.75g bid	0.5g bid	Monitor level twice or triple weekly
Sirolimus	Loading dose 6mg/d, maintenance dose 2mg/d	Loading dose 6mg/d, maintenance dose 1mg/d	Monitor level twice or triple weekly
Drug	Creatinine clearance rate	Recommended adjustment	Recommended monitoring
Cyclosporine A	Ccr of 20-50ml/min	Dose as in normal renal function	Monitor level once or twice weekly
	Ccr of 10-20ml/min		
	Ccr<10ml/min		
Tacrolimus	Ccr of 20-50ml/min	Dose as in normal renal function	Monitor level once or twice weekly
	Ccr of 10-20ml/min		
	Ccr<10ml/min		
Mycophenolate	Ccr of 25-50ml/min	Dose as in normal renal function	Monitor level once or twice weekly
	Ccr of 10-25ml/min	Mycophenolate mofetil: 1 g twice a day, starting immediately post- transplant. Mycophenolate sodium: Maximum1440 mg daily, starting immediately post-transplant.	Monitor level twice or triple weekly
	Ccr<10ml/min	Mycophenolate mofetil: 1 g twice a day, starting immediately post-transplant. Mycophenolate sodium: Maximum1440 mg daily, starting immediately post-transplant.	Monitor level twice or triple weekly
Sirolimus	Ccr of 20-50ml/min	Dose as in normal renal function	Monitor level once or twice weekly
	Ccr of 10-20ml/min		
	Ccr<10ml/min		

**Table 2:** Immunosuppressants dose adjustment according to routine, pharmacogenetics and combination medications.

Trough concentration	Recommended adjustment	Timing of next level	
30% below goal	Increase dose by 0.25	4–7 days	
50% below goal	Increase dose by 0.5	4–7 days	
>50%-100% below goal	Increase dose by 1	2–3 days	
>200% below goal	Load with double dose once then increase dose by 0.5	2–3 days	
30% above goal	Decrease dose by 0.25	4–7 days	
50% above goal	Decrease dose by 0.5	4–7 days	
>50%-100% above goal	Hold one dose then decrease dose by 0.25-0.5	2–3 days	
>200% above goal	Drug withdrawal then increase dose by 0.5	Daily	
Drug	CYP genotype	Standard recommended dose(mg/kg)	Recommended adjustment
Tacrolimus	CYP3A5*3/*3	0.15 mg/kg/day	0.1-0.15 mg/kg/day
	СYР3А5*1/*3	0.15 mg/kg/day	1.5-2 times the standard dose but do not exceed 0.3 mg/kg/day
	CYP3A5*1/*1	0.15 mg/kg/day	1.5-2 times the standard dose but do not exceed 0.3 mg/kg/day
	CYP3A5*3/*3 + CYP3A4*22	0.15 mg/kg/day	0.14 mg/kg/day
	CYP3A5*3/*3 + CYP3A4*1/*1	0.15 mg/kg/day	0.2-0.25 mg/kg/day
	CYP3A5*1 + CYP3A4*1/*1	0.15 mg/kg/day	0.3-0.4 mg/kg/day
	SLCO1B3 334G	0.15 mg/kg/day	0.14 mg/kg/day or lower
	SLCO1B3 699A	0.15 mg/kg/day	0.14 mg/kg/day or lower
Cyclosporine	CYP3A5*1	6mg/kg/day	6.5-7mg/kg/day or higher
	CYP3A4*1B	6mg/kg/day	6.5-7mg/kg/day or higher
	CYP3A5*3	6mg/kg/day	5mg/kg/day or lower
	CYP3A4*1/*1	6mg/kg/day	6.5-7mg/kg/day or higher
Mycophenolic acid	MRP2-24T>C	1.5g/day	2g/day or higher
	UGT1A9-440C>T	1.5g/day	2g/day or higher
	UGT2B7-900A>G	1.5g/day	2g/day or higher
	UGT2B7 IVS1+985AG	1.5g/day	1g/day or lower
	UGT1A9-1818CT	1.5g/day	1g/day or lower
	UGT1A9-440C>T	1.5g/day	1g/day or lower
	UGT1A9-331T>C	1.5g/day	1g/day or lower
	UGT1A8*2	1.5g/day	2g/day or higher
	UGT1A7 622TT	1.5g/day	2g/day or higher
	UGT1A9 1399 T/T	1.5g/day	1g/day or lower
	UGT1A9 T-275A	1-1.5g/day	2g/day or higher
	UGT1A9 C-2152T	1-1.5g/day	2g/day or higher
Sirolimus	CYP3A5*1/*3	Loading dose 6mg/day Maintenance dose 2mg/day	Maintenance dose 2.5mg/day
	CYP3A5*1/*1	Loading dose 6mg/day Maintenance dose 2mg/day	Maintenance dose 2.5mg/day
	ABCB1 3435 C/T	Loading dose 6mg/day Maintenance dose 2mg/day	Maintenance dose 1.5mg/day
	CYP3A4 C/C	Loading dose 6mg/day Maintenance dose 2mg/day	Maintenance dose 1.5mg/day
Concomitant medication	Recommended adjustment	Inpatient	Outpatient
Strong CYP3A4 inhibitor	Decrease dose by 50%-70%	Daily levels	Levels 2–3 times weekly
◼ Posaconazole			
◼ Voriconazole			
Moderate CYP3A4 inhibitor	Continue current dose	Levels Monday, Wednesday, Friday	Levels 1–2 times weekly
◼ Amiodarone			
◼ Diltiazem			
◼ Fluconazole			
◼ Grapefruit juice			
◼ Isavuconazonium			
◼ Letermovir			
◼ Schisandra			
◼ Verapamil			
Strong CYP3A4 inducers	Increase dose by 25–50%	Daily levels	Levels 2–3 times weekly
◼ Carbamazepine			
◼ Phenytoin			
◼ Phenobarbital			
◼ Primidone			
◼ Rifampin			
Moderate CYP3A4 inducers	Increase dose by 25% or continue current dose	Levels Monday, Wednesday, Friday	Levels 1–2 times weekly
◼ Bosentan			
◼ Dexamethasone			
◼ Rifabutin			
◼ Rifapentine			
◼ St. John's wort			

**Figure 2:**
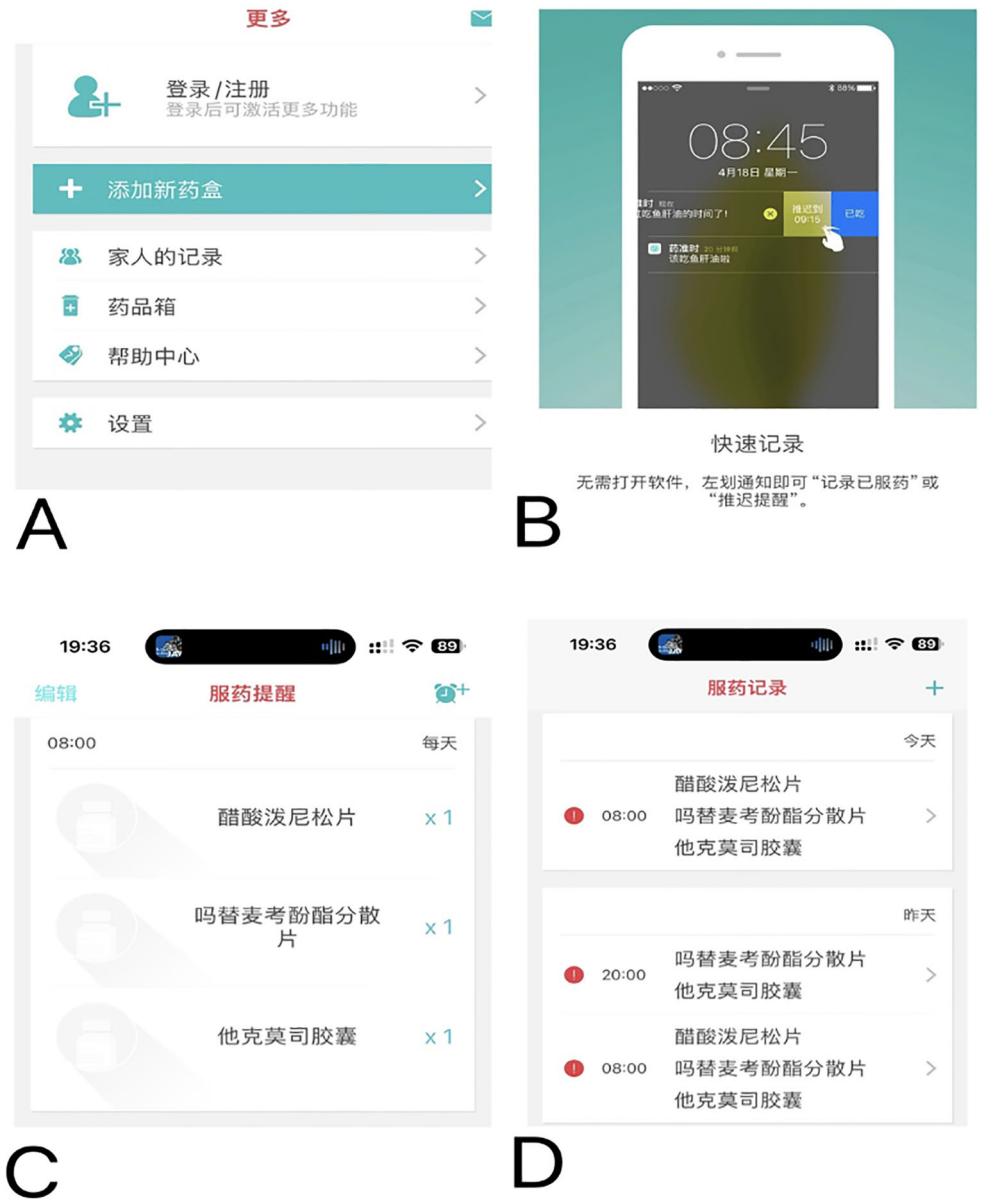
Screenshots of the home-based monitoring component of the application.

**Figure 3:**
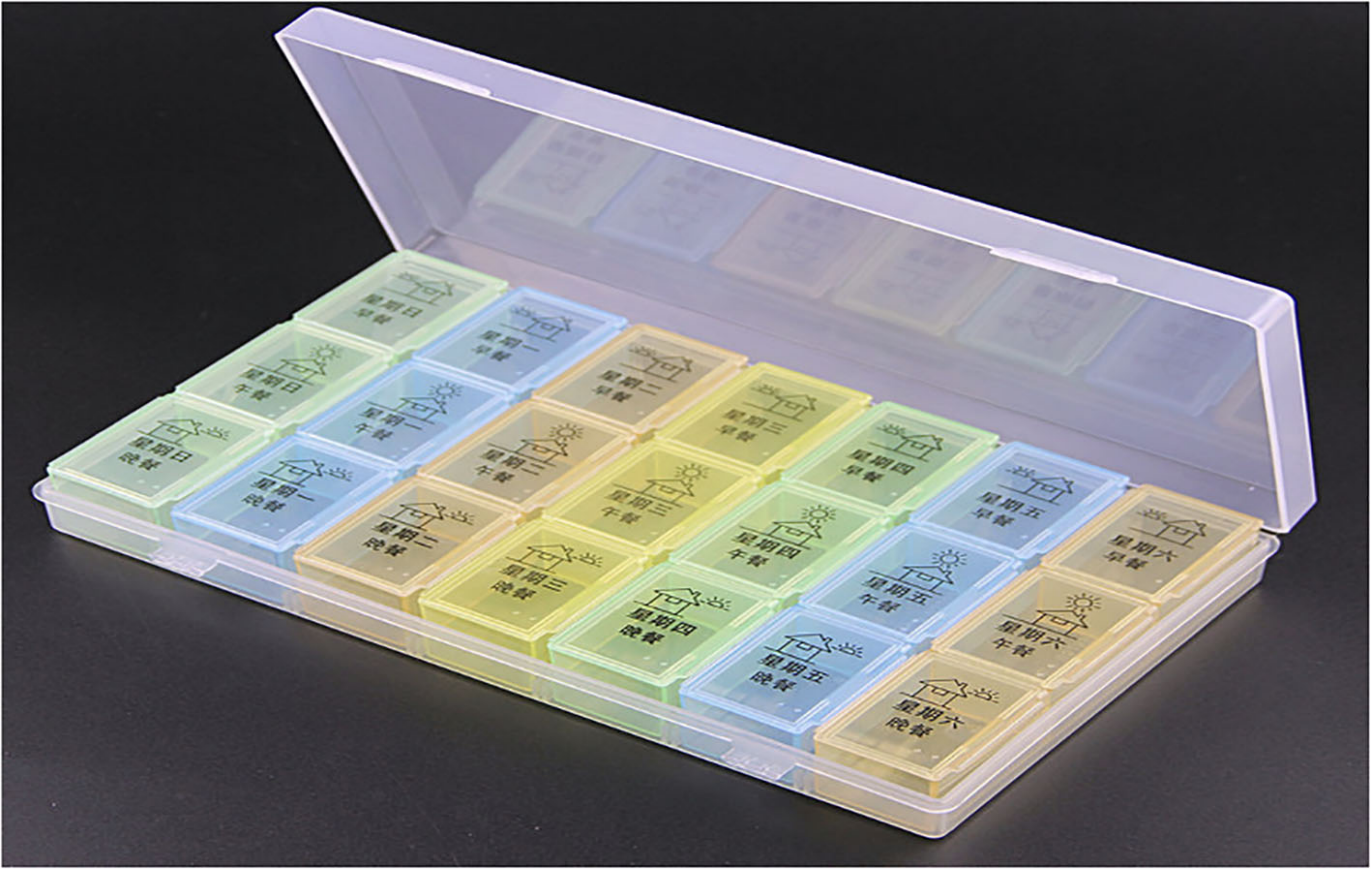
The pill box designed by pharmacists with reminder labels but without automatic dose alerts.

The medication education takes place on the day of discharge (or the day before if the discharge is scheduled), after a brief reminder of the instructions/information, a list of warnings and precautions will be issued to the patient. For each drug, the patient had to repeat in their own words its goal and moment to be taken relative to the time, other medications, and/or meals. In addition to usual education, the low adherence (MA-VAS < 6 points) and medium adherence (MA-VAS 6 to < 8 points) patients received clinical pharmacist-led supplemental medication therapy monitoring and management, utilizing a smartphone-enabled mobile application (**Figure 2**), integrated with a pill box with a reminder label (**Figure 3**). High-adherence patients make their own decisions on whether or not to use the app and pill box.

For the home-based monitoring component of the application, pharmacists updated discharge medication information for patients (**Figure 2A**); this includes inputting the type and name of medication, setting the times when medications are taken (push notifications via smartphone), setting reminders of medication taking, and setting medication adverse effects and their severity monitoring at least once a month or more frequently if desired. When the patient taps the “Medications” button on the home screen (**Figure 2B**), he or she can review the complete medication list (**Figure 2C**). In addition, patients can review their medication regimen and scheduled administration times and select which medications they are taking to document self-reported adherence (**Figure 2D**). From this application, they can also contact the transplant center or pharmacist for consultation and send real-time message alerts to the pharmacist to document a medication change, make an outpatient appointment, or hospital admission.

### 2.3 Outcome assessment

After kidney transplantation, blood samples were collected every 1~2 days or as required by clinical treatment. The blood concentration of immunosuppressants were monitored after the drug reached a steady state. After the patients were given immunosuppressants for 12h, 1 ~ 2ml peripheral venous blood was collected and placed in the EDTA anticoagulation tube at 7:30 am on a fasting state, and the blood samples were placed in the −20 °C refrigerator. The whole blood trough concentration of immunosuppressants were determined by the Thermo Scientific™ TSQ Endura™. The blood samples were all taken under the same conditions and analyzed in the same laboratory (Biological Pharmacology, People’s Hospital of Guangxi Zhuang Autonomous Region), thus eliminating interlaboratory variability. The target CsA C_min_ was defined as 150–300 ng/ml during the first month (M1) post-transplantation, 150–250 ng/ml during the second (M2) and third (M3) months, and 80–120 ng/ml one year and thereafter (15). The target TAC C_min_ was 8–12 ng/ml during M1, 6–10 ng/ml during M2 and M3, and 4–8 ng/ml one year and afterward (16–18). The C_min_ of MMF was defined as the plasma concentration of its active metabolite mycophenolic acid (MPA) measured immediately prior to drug administration. The long-term post-transplant target C_min_ range was established as 1–3.5 mg/ml (19). SRL was recommended to be started from M3 with a target defined as 4–7 ng/ml (20). The primary outcome of this study was the %CV of immunosuppressants. %CV was calculated as (Standard Deviation of C_min_/Mean of C_min_) × 100%, was calculated as a measure of the intraindividual variability. *C_i_* was defined as the single-point serum drug concentration measurement, and *n* was defined as the number of measurements.


$$\%CV=\left(\frac{SD}{Mean}\right)100$$

$$SD=\sqrt{\frac{\sum_{i=1}^{n}{\left(C_{i}-Mean\right)}^{2}}{n-1}}$$

$$Mean=\frac{\sum_{i=1}^{n}C_{i}}{n}$$

A C_min_ %CV below 22% was defined as low variability, a value between 22% and 30% was defined as intermediate variability, and a value above 30% was defined as high variability, as reported previously (21). Secondary outcomes included serum creatinine, urea, cystatin C (serum biomarkers of glomerular filtration rate) and urinary microalbumin (an early indicator of glomerular endothelial injury).

### 2.4 Data collection

Baseline characteristics collected included demographic information, education level, type of primary nephropathy, comorbidities, length of hospital stay, and death during the study period. Missing data were obtained through direct communication with the patients and their families, as well as with physicians responsible for the patient’s treatment. The data were collected retrospectively from the electronic medical record and crosschecked by two independent investigators. To avoid detection bias, the clinical pharmacist participating in the protocol could not assess the outcome.

### 2.5 Statistical analysis

Data were presented as means and SDs or medians and interquartile range for continuous variables and frequencies and percentages for categorical variables. Comparisons between protocol groups were run with a 2-sample t test or Wilcoxon rank sum test or a chi-square test or Fisher’s exact test, where appropriate. The statistical difference for significance was set at *P*<0.05 in all cases. All tests were conducted with SPSS 29.0 software.

## 3 Results

### 3.1 Clinical basic information

**Figure 4:**
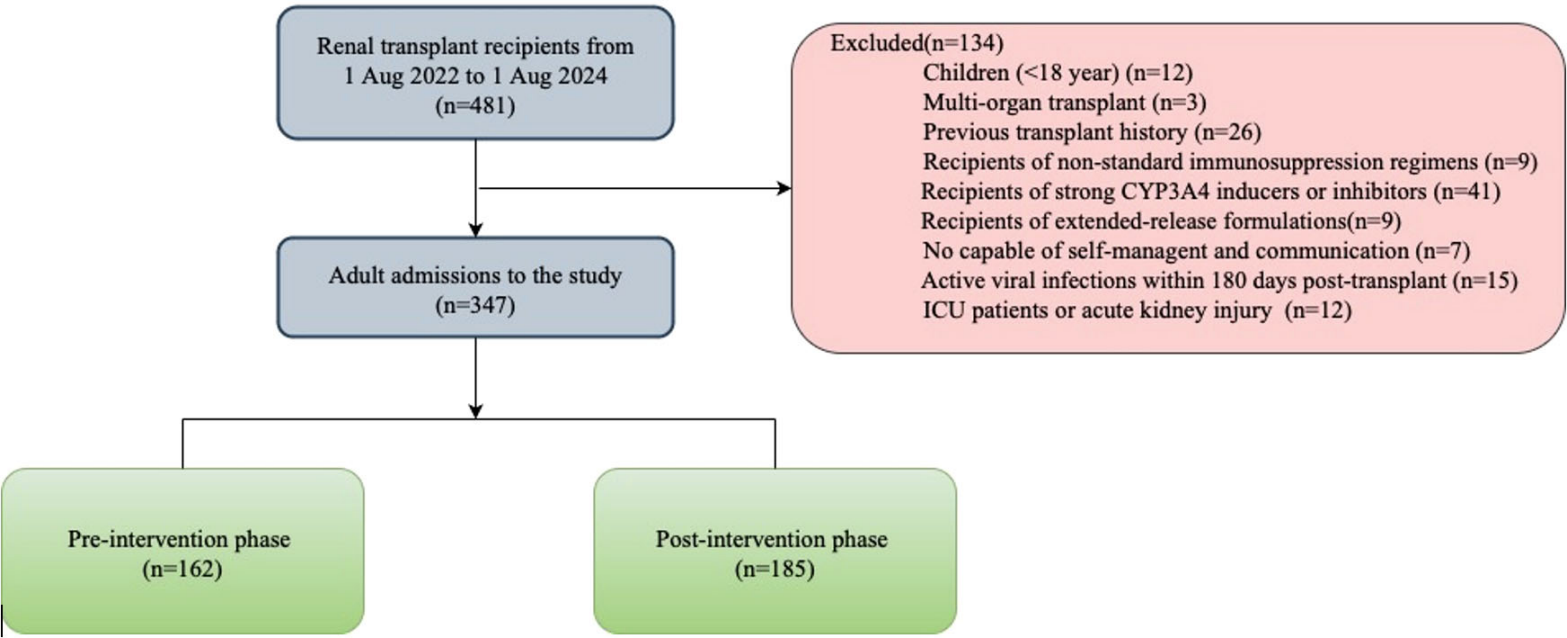
Flowchart of study patients.

Participant screening and selection were outlined in **Figure 4**. Of 481 patients who underwent kidney transplantation during the prespecified timeframe, 347 were enrolled. These comprised the pre-intervention (n=162) and post-intervention (n=185) groups. The baseline characteristics for these patients were similar between the groups. There was no significant difference between the two groups in the following: education level, etiology of end-stage renal disease, comorbidities, and the average length of stay during the study period (**Table 3**). Patients were followed for 12 months from their date of transplant.

**Table 3:** Demographic and baseline clinical characteristic of the study participants.

Characteristic	Control (n=162)	Intervention (n=185)	*P*-value
Age, y	43.8±16.2	39.1±10.3	0.912
Male (%)	116 (71.6%)	112 (62.2%)	0.883
BMI (kg/m2)	23.1±3.2	22.5±4.2	0.679
Systolic Blood Pressure (mm Hg)	143.49 ±27.3	135.56±32.1	0.491
Diastolic blood pressure (mm Hg)	85.31±15.52	82.63±13.15	0.745
Heart rate (beat/min)	82.17 ±10.9	82.65 ±11.2	>0.997
Education level			
Primary and below	5	4	>0.999
Secondary school	56	63	0.857
College and above	101	113	0.811
Aetiology of end-stage renal disease			
Diabetic nephropathy, hypertensive nephropathy, systemic or autoimmune glomerulonephritis, chronic interstitial nephropathy	99 (61.1%)	117 (65.0%)	0.893
Genetic renal disease (polycystic kidney disease)	5 (3.1%)	8 (4.4%)	0.742
Other causes (obstructive uropathy, iatrogenesis, occupational exposure or haematologic disease)	11 (6.8%)	16 (8.9%)	0.591
Not determined	47 (29.0%)	39 (21.7%)	0.837
Comorbidities (pre-transplantation)			
Diabetes	77 (47.5%)	89 (49.4%)	0.493
Hypertension	96 (59.2%)	121 (67.4%)	0.239
Deep vein thrombosis	13 (8.0%)	15 (8.3%)	0.626
Chronic obstructive pulmonary disease	32 (19.8%)	33 (18.3%)	0.735
Length of hospital stay, mean±SD	25.4±8	24.3±7	0.763

### 3.2 Improvement of immunosuppressants reaching the target concentration ranges

#### 3.2.1 TAC

The analysis of TAC C_min_ relative to the target range revealed significant differences between pre- and post-intervention groups (**Table 4**). Over the study period, a total of 23,791 measurements were recorded (11,869 pre-intervention; 11,922 post-intervention). For each month, the post-intervention group consistently exhibited a higher proportion of patients within the target range compared to the pre-intervention group (*P*=0.013). In contrast to the pre-intervention group, the post-intervention cohort demonstrated sustained reductions in the proportion of patients with out-of-range (*P*=0.021). Overall, the post-intervention group achieved a significantly higher proportion of TAC C_min_ within the target range 72.4% (8,631/11,922) compared to the pre-intervention group 58.3% (6,919/11,869; *P*=0.012). Concurrently, the proportions of measurements above and below the target range decreased post-intervention: above-target values declined from 19.0% (2,255/11,869) to 11.6% (1,383/11,922), and below-target values decreased from 22.7% (2,695/11,869) to 16.0% (1,908/11,922).

**Table 4:** C_min_ of TAC above, within and below the target range.

Months	Groups	The number of patients				
		Within the target range (%)	*P* value	Outside the target range (%)	*P* value	
1	Pre-intervention (n=127)	61.4	0.013	38.6	0.021	
	Post-intervention (n=148)	81.8		18.2		
2	Pre-intervention (n=131)	58.8		41.2		
	Post-intervention (n=149)	71.8		28.2		
3	Pre-intervention (n=131)	64.9		35.1		
	Post-intervention (n=149)	75.8		24.2		
4	Pre-intervention (n=130)	55.4		44.6		
	Post-intervention (n=149)	110		39		
5	Pre-intervention (n=130)	59.2		40.8		
	Post-intervention (n=149)	77.2		22.8		
6	Pre-intervention (n=130)	56.2		43.8		
	Post-intervention (n=149)	73.2		26.8		
7	Pre-intervention (n=130)	61.5		38.5		
	Post-intervention (n=149)	81.2		18.8		
8	Pre-intervention (n=130)	60.0		40.0		
	Post-intervention (n=149)	84.6		15.4		
9	Pre-intervention (n=130)	54.6		45.4		
	Post-intervention (n=149)	79.9		20.1		
10	Pre-intervention (n=130)	66.9		33.1		
	Post-intervention (n=149)	83.2		16.8		
11	Pre-intervention (n=130)	60.8		39.2		
	Post-intervention (n=149)	87.9		12.1		
12	Pre-intervention (n=130)	63.8		36.2		
	Post-intervention (n=149)	83.9		16.1		
Groups		Within the target range, n (%)	Above the target range, n (%)	Below the target range, n (%)	Total number of measurements	*P* value
Pre-intervention		6919 (58.3%)	2255 (19.0%)	2695 (22.7%)	11869 (100%)	0.012
Post-intervention		8631 (72.4%)	1383 (11.6%)	1908 (16.0%)	11922 (100%)	

#### 3.2.2 CsA

Differences in CsA C_min_ were also observed between the pre- and post-intervention periods (**Table 5**). Across all months, the post-intervention group maintained a significantly higher proportion of patients within the target range relative to the pre-intervention group (*P*=0.038). Conversely, the post-intervention cohort displayed persistent decreases in the proportion of patients with concentrations out-of-range compared to pre-intervention levels (*P*=0.025). Over the study period, a total of 10,452 measurements (pre-intervention: 5,238; post-intervention: 5,214) were recorded. Post-intervention, a significant improvement in target adherence was observed, with 63.7% of measurements falling within the target range compared to 46.5% pre-intervention (*P*=0.037). Concurrently, the proportion of values above the target range decreased from 21.7% to 13.0%, while those below the target range decreased from 31.8% to 23.3%.

**Table 5:** C_min_ of CsA above, within and below the target range.

Months	Groups	The number of patients				
		Within the target range (%)	*P* value	Outside the target range (%)	*P* value	
1	Pre-intervention (n=35)	54.3	0.038	45.7	0.025	
	Post-intervention (n=37)	81.1		18.9		
2	Pre-intervention (n=31)	58.1		41.9		
	Post-intervention (n=36)	75.0		25.0		
3	Pre-intervention (n=31)	51.6		48.4		
	Post-intervention (n=36)	80.6		19.4		
4	Pre-intervention (n=32)	43.8		56.2		
	Post-intervention (n=36)	77.8		22.2		
5	Pre-intervention (n=32)	53.1		46.9		
	Post-intervention (n=36)	86.1		13.9		
6	Pre-intervention (n=32)	40.6		59.4		
	Post-intervention (n=36)	69.4		30.6		
7	Pre-intervention (n=32)	53.1		46.9		
	Post-intervention (n=36)	80.6		19.4		
8	Pre-intervention (n=32)	43.8		56.2		
	Post-intervention (n=36)	72.2		27.8		
9	Pre-intervention (n=32)	56.2		43.8		
	Post-intervention (n=36)	77.8		22.2		
10	Pre-intervention (n=32)	46.9		53.1		
	Post-intervention (n=36)	61.1		38.9		
11	Pre-intervention (n=32)	59.4		40.6		
	Post-intervention (n=36)	75.0		25.0		
12	Pre-intervention (n=32)	56.3		43.7		
	Post-intervention (n=36)	80.6		19.4		
Groups		Within the target range, n (%)	Above the target range, n (%)	Below the target range, n (%)	Total number of measurements	*P* value
Pre-intervention		2436 (46.5%)	1137 (21.7%)	1665 (31.8%)	5238 (100%)	0.037
Post-intervention		3321 (63.7%)	678 (13.0%)	1215 (23.3%)	5214 (100%)	

#### 3.2.3 MMF

The analysis of MMF C_min_ across pre- and post-intervention groups revealed significant changes in the proportion of patients within the target therapeutic range (**Table 6**). In the post-intervention group demonstrated a higher number of patients achieving MMF levels within the target range compared to the pre-intervention group across all 12 months (*P*=0.012). In contrast to pre-intervention levels, the post-intervention group demonstrated sustained reductions in the proportion of patients exhibiting non-target drug concentrations (*P* =0.033). In the pre-intervention group, 65.3% (5,565/8,523) of MMF C_min_ measurements fell within the target range, whereas post-intervention, this proportion increased to 76.0% (6,490/8,540; *P*=0.025). The proportion of above the target range decreased from 18.5% (1,577) pre-intervention to 13.7% (1,170) post-intervention, while below the target range declined from 16.2% (1,381) to 10.3% (880).

**Table 6:** C_min_ of MMF above, within and below the target range.

Months	Groups	The number of patients				
		Within the target range (%)	*P* value	Outside the target range (%)	*P* value	
1	Pre-intervention (n=162)	51.2	0.012	48.8	0.033	
	Post-intervention (n=185)	70.3		29.7		
2	Pre-intervention (n=162)	54.3		45.7		
	Post-intervention (n=185)	77.8		22.3		
3	Pre-intervention (n=162)	55.6		44.4		
	Post-intervention (n=185)	75.1		24.9		
4	Pre-intervention (n=136)	49.3		50.7		
	Post-intervention (n=164)	71.9		28.1		
5	Pre-intervention (n=136)	62.5		37.5		
	Post-intervention (n=164)	74.4		25.6		
6	Pre-intervention (n=139)	50.4		49.6		
	Post-intervention (n=160)	83.8		16.2		
7	Pre-intervention (n=139)	56.8		43.2		
	Post-intervention (n=160)	80.0		20.0		
8	Pre-intervention (n=139)	58.3		41.7		
	Post-intervention (n=160)	81.3		18.7		
9	Pre-intervention (n=139)	59.7		40.3		
	Post-intervention (n=160)	71.2		28.8		
10	Pre-intervention (n=142)	52.8		47.2		
	Post-intervention (n=164)	78.7		21.3		
11	Pre-intervention (n=142)	51.4		48.6		
	Post-intervention (n=164)	71.9		28.1		
12	Pre-intervention (n=142)	55.6		44.4		
	Post-intervention (n=164)	76.8		23.2		
Groups		Within the target range, n (%)	Above the target range, n (%)	Below the target range, n (%)	Total number of measurements	*P* value
Pre-intervention		5565 (65.3%)	1577 (18.5%)	1381 (16.2%)	8523 (100%)	0.025
Post-intervention		6490 (76.0%)	1170 (13.7%)	880 (10.3%)	8540 (100%)	

#### 3.2.4 SRL

The analysis of SRL C_min_ demonstrated significant improvements in the proportion of patients within the therapeutic target range following the intervention (**Table 7**). The post-intervention group consistently exhibited higher rates of SRL levels within the target range compared to the pre-intervention group across all months (*P*=0.048). The post-intervention group exhibited persistent declines in the proportion of patients with concentrations deviating from the therapeutic target range compared to pre-intervention levels (*P*=0.045). Overall, the pre-intervention group, 51.9% (658/1,269) of measurements were within the target range, which increased substantially to 80.2% (1,007/1,255) post-intervention (*P*=0.018). Concurrently, the proportion of measurements above the target range decreased from 16.9% (215) to 5.98% (75), and those below the target range declined from 31.2% (396) to 13.8% (173).

**Table 7:** C_min_ of SRL above, within and below the target range.

Months	Groups	The number of patients				
		Within the target range (%)	*P* value	Outside the target range (%)	*P* value	
4	Pre-intervention (n=26)	46.2	0.048	53.8	0.045	
	Post-intervention (n=21)	95.2		4.8		
5	Pre-intervention (n=26)	53.8		46.2		
	Post-intervention (n=21)	80.1		23.8		
6	Pre-intervention (n=23)	52.2		47.8		
	Post-intervention (n=25)	84.0		16.0		
7	Pre-intervention (n=23)	47.8		52.2		
	Post-intervention (n=25)	80.0		20.0		
8	Pre-intervention (n=23)	43.5		56.5		
	Post-intervention (n=25)	76.0		24.0		
9	Pre-intervention (n=23)	56.5		43.5		
	Post-intervention (n=25)	88.0		12.0		
10	Pre-intervention (n=20)	55.0		45.0		
	Post-intervention (n=21)	85.7		14.3		
11	Pre-intervention (n=20)	75.0		25.0		
	Post-intervention (n=21)	90.5		9.5		
12	Pre-intervention (n=20)	60.0		40.0		
	Post-intervention (n=21)	81.0		19.0		
Groups		Within the target range, n (%)	Above the target range, n (%)	Below the target range, n (%)	Total number of measurements	*P* value
Pre-intervention		658 (51.9%)	215 (16.9%)	396 (31.2%)	1269 (100%)	0.018
Post-intervention		1007 (80.2%)	75 (5.98%)	173 (13.8%)	1255 (100%)	

### 3.3 Improvement of immunosuppressant CV

#### 3.3.1 TAC

The TAC CV in the first 3 months in the pre-intervention group was 18.28% compared with 8.92% in the post-intervention group (*P*=0.031; **Table 8**), From months 3-6, the TAC CV was 10.01% in the pre-intervention group versus 21.98% in the post-intervention group (*P*=0.018; **Table 8**). During the 6–12 months post-transplantation, the TAC CV was 21.28% in the pre-intervention group and 11.67% in the post-intervention group (*P*=0.038; **Table 8**).

**Table 8:** CV of TAC, CsA, MMF, SRL.

Variable	Pre-intervention (n=162)	Post-intervention (n=185)	*P* value
TAC 3 mo after transplant	18.28%	8.92%	0.031
TAC 3-6 mo after transplant	21.98%	10.01%	0.018
TAC 6-12 mo after transplant	21.28%	11.67%	0.038
CsA 3 mo after transplant	22.97%	7.14%	0.004
CsA 3-6 mo after transplant	27.77%	14.83%	0.032
CsA 6-12 mo after transplant	33.38%	16.77%	0.047
MMF 3 mo after transplant	56.22%	34.84%	0.148
MMF 3-6 mo after transplant	49.26%	39.92%	0.510
MMF 6-12 mo after transplant	51.54%	51.68%	0.993
SRL 3-6 mo after transplant	27.83%	22.30%	0.488
SRL 6-12 mo after transplant	26.05%	17.02%	0.194

#### 3.3.2 CsA

During the first 3 months post-transplantation, the CsA CV was 22.97% in the pre-intervention group compared to 7.14% in the post-intervention group (*P*=0.004; **Table 8**);. At 3–6 months post-transplantation, the CsA CV were 27.77% (pre-intervention) versus 14.83% (post-intervention) (*P*=0.032; **Table 8**). From 6 to 12 months post-transplantation, the CsA CV in the pre-intervention group was 33.38%, while the post-intervention group exhibited a value of 16.77% (*P*=0.047; **Table 8**).

#### 3.3.3 MMF

In the initial 3-month period post-transplantation, the CV of MMF was 56.22% in the pre-intervention group versus 34.84% in the post-intervention group (*P*=0.148; **Table 8**). Between 3 to 6 months post-transplantation, the MMF CV presented 49.26% (pre-intervention) and 39.92% (post-intervention) (*P*=0.510; **Table 8**). Subsequently, from 6 to 12 months post-transplantation, the pre-intervention group showed a CV of 51.54%, whereas the post-intervention group demonstrated a value of 51.68% (*P*=0.993; **Table 8**).

#### 3.3.4 SRL

Between 3 to 6 months post-transplantation, the SRL CV was 27.83% in the pre-intervention group compared to 22.30% in the post-intervention group (*P*=0.488; **Table 8**). From 6 to 12 months post-transplantation, the SRL CV presented in the pre-intervention group at 26.05%, whereas the post-intervention group demonstrated a value of 17.02% (*P*=0.194; **Table 8**).

#### 3.3.5 Overall trends in variability of immunosuppressants

During the 0–3 months post-transplantation, in the pre-intervention group, the CV for TAC and CsA were both below 30% (indicating moderate to low variability), whereas the CV for MMF exceeded 30% at 56.22% (high variability). In the post-intervention group during the first 3 months, the CVs for TAC and CsA remained below 22% (low variability).

At 3–6 months post-transplantation, in the pre-intervention group, the CVs for CsA, MMF, and SRL all exceeded 22% (moderate to high variability), except for TAC. In contrast, the post-intervention group exhibited low variability for TAC and CsA, with CVs of 10.01% and 16.83%, respectively. MMF in this group showed high variability (CV 39.92%), while SRL demonstrated moderate variability (CV 22.30%).

At 6–12 months post-transplantation, in the pre-intervention group, CVs of CsA, MMF, and SRL revealed above 22% (moderate to high variability), while TAC presented low variability. In the post-intervention group, MMF maintained high variability (CV 51.68%), while TAC, CsA, and SRL all displayed low variability.

### 3.4 Improvement of transplanted kidney function

**Figure 5:**
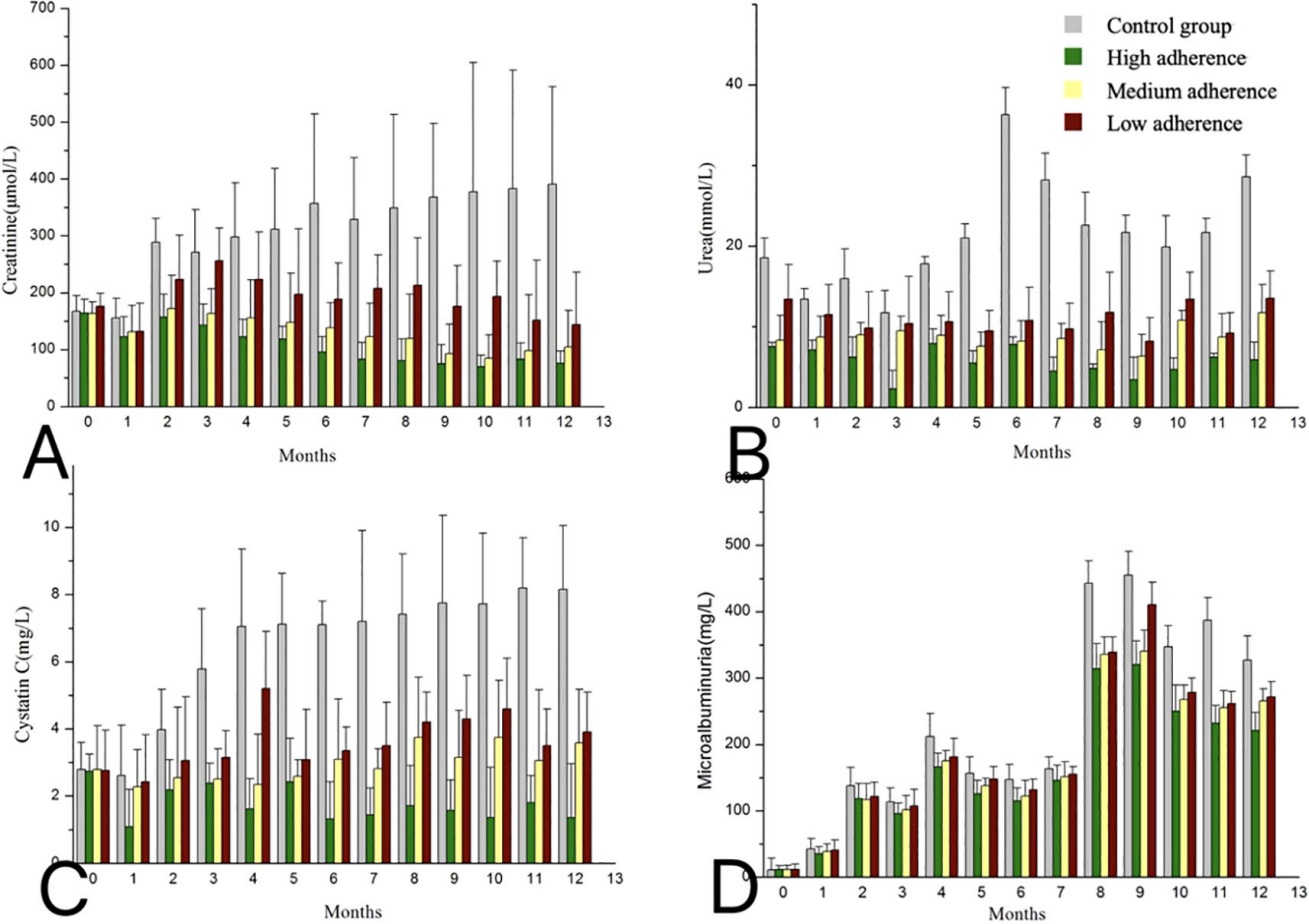
Changes in creatinine **(A)**, urea **(B)**, cystatin C **(C)**, and microalbuminuria **(D)** between pre-intervention and post-intervention in patients with different levels of compliance within one year after transplantation. Results are presented as the means± SE.

To substantiate the effect of measures for the patients with different medication adherence, we further examined renal function-associated indicators, including serum creatinine, urea, cystatin C, and microalbuminuria. From 2 months to one year after transplant, serum creatinine and cystatin C were significantly lower in the post-intervention group compared to pre-intervention patients (*P* < 0.05; **Figures 5A, C**). After the implementation of the measures, the urea level in the post-intervention group was always lower than that in the pre-intervention group, and this difference was particularly obvious in 6 months after transplant (*P* < 0.05; **Figure 5B**). No difference in microalbuminuria was found between the pre-intervention and post-intervention groups within 7 months after transplant; however, microalbuminuria increased significantly in the pre-intervention group from 8 months and later (*P* < 0.05; **Figure 5D**).

## 4 Discussion

In this study, we describe how pharmacists developed and implemented PLIS at a general hospital with a transplantation center. Pharmacists’ expertise is valuable for the care of patients who have undergone renal transplant, which requires close monitoring and adjusting of complex, frequently changing medication regimens to ensure survival of the transplanted graft. In addition, pharmacist management is closely associated with patient adherence to immunosuppressive therapy, which is also a crucial factor in ensuring post-transplant graft survival. Integrating pharmacists with this method into the center shifted workflow to allow pharmacists more responsibilities for managing immunosuppressant regimens and recipients’ adherence. Our findings demonstrate that the PLIS can be implemented, measured, and evaluated methodologically to support program sustainability.

Existing research predominantly has documented pharmacists’ implementation of diverse interventions, including manual techniques and mobile health technologies, within the inpatient or outpatient (22–25). While these studies have yielded certain positive outcomes, they often lacked involvement in collaborative dose adjustments with physicians, as well as post-discharge medication guides and adherence monitoring. Furthermore, there was a lack of effective strategies to enhance the various factors mentioned above that impact patient medication adherence. This gap contributed to suboptimal treatment efficacy for patients. PLISs extended pharmacists’ scope of practice, allowing pharmacists more autonomy and involvement in helping patients achieve long-term graft management goals. Furthermore, PLISs incorporated additional programmatically driven opportunities for intervention that may further improve outcomes.

The implementation of this practice achieved effectiveness from the start, largely due to pharmacists’ predictive adherence to immunosuppressants in outpatient pretransplantation. Numerous factors contributing to medication non-compliance have been elucidated, spanning socioeconomic determinants (e.g., age, gender, social support networks, employment status, level of education), patient-centric aspects (such as health literacy, personal beliefs, concurrent morbidities, and substance dependencies), disease-specific variables (e.g., time post-transplantation, history of transplant surgeries, donor source-living or cadaveric, presence of complications), treatment-related issues (including medication regimen complexity, dosing frequency, incidence of adverse effects, polypharmacy), and healthcare system-associated factors unique to different national contexts (26). This initial step involved the stratification of candidate patients, thereby fulfilling multiple objectives: it enabled the identification of individuals with suboptimal adherence within the substantial renal transplant candidates. Subsequently, this process facilitated precise categorization and tagging, which in turn informed the development of tailored intervention strategies for enhanced management and outcomes in subsequent stages of care.

In the pre-intervention group, adjustments of immunosuppressants during hospitalization were primarily based on blood concentration levels and the physician’s routine clinical expertise. Variability in laboratory methodologies and protocols contributed to disparities in the precision of blood concentration determination outcomes (27, 28). This inconsistency was further exacerbated by differences in clinical experience among physicians and the use of distinct target concentrations across various centers (29, 30). Conversely, within the post-intervention group, the involvement of specialist pharmacists in tailoring and modulating the recipients’ immunosuppressive regimen adhered strictly to principles governing liver and kidney function (31), pharmacogenomics (32), and drug-drug interactions (33), thereby significantly mitigating such discrepancies. During our initial pilot, pharmacists had impacted immunosuppressive management not only for transplant patients in one department but also for additional patients with immune diseases, such as lupus nephritis, suggesting potential patient benefits from this approach (34).

Despite the efficacy of standardized pharmacist management during hospitalization in ensuring patient medication adherence, the post-discharge standardized utilization of immunosuppressive therapies presents significant challenges, particularly among patients exhibiting MMAS scores of 8 or below. To address this issue, pharmacists endeavor to enhance compliance by implementing pre-discharge medication education programs targeted at these patients. Additionally, for those classified as having low-to-medium adherence, supplementary interventions include pill boxes containing daily identification labels and facilitating the installation of mobile applications designed to serve as medication reminder systems. The findings revealed that patients receiving standard and adjunctive pharmacist-led interventions exhibited a significantly higher proportion of individuals achieving C_min_ within the therapeutic target range and a reduced incidence of out-of-range C_min_ compared to the pre-intervention group. Furthermore, analysis of total measurements indicated that the post-intervention group demonstrated an increased frequency of C_min_ within the target range and a concurrent decrease in out-of-range measurements compared to pre-intervention, with these differences demonstrating statistical significance. Taken as a whole, these results suggest that the full integration of a pharmacist into the transplantation team (the pharmacist’s role was to validate prescriptions, educate patients, perform medication reconciliation, monitor C_min_, and suggest adjustments, provide adjunctive interventions) helped to maintain the C_min_ in the target range during the patient’s hospital stay and the post-discharge period. Previous studies have reported various pharmacist-led medication management interventions for renal transplant recipients, demonstrating corresponding positive outcomes (25, 35–38). However, these interventions were typically unimodal or focused on isolated aspects such as medication non-adherence. Our study fundamentally differs by implementing a comprehensive care continuum that integrates personalized dosing regimens, adherence assessment, adherence-enhancing education, and practical tools (including customized medication organizers and mobile health technologies) across all treatment phases (outpatient and inpatient settings). In China, pharmacist-led clinics and inpatient pharmaceutical services generally operate independently, with limited workflows for longitudinal patient follow-up. This results in fragmented medication management. Our integrated approach not only delivered optimized pharmaceutical care for transplant recipients but also aligned with China’s pharmacy service transformation policy to advance clinical pharmacy ambulatory service development.

Current literature presents divergent evidence regarding the relationship between medication adherence and CV. Multiple studies indicate that implementation non-adherence correlates with higher CV or time-weighted CV alongside reduced tacrolimus blood concentrations (39, 40). Conversely, the nonadherent behavior—measured through electronic monitoring or self-report—does not significantly affect intra-patient variability (IPV) (41). TAC IPV group classification fails to consistently correlate with behavioral adherence measures, potentially leading to misidentification of nonadherence in adolescent and young adult kidney transplant recipients (42). While females demonstrate better self-reported adherence than males, they exhibit greater tacrolimus level variability (43). Furthermore, high intrapatient tacrolimus variability may associate with elevated donor-derived cell-free DNA during the first post-transplant year (44). A meta-analysis of 15 randomized controlled trials indirectly supports this complexity, identifying genotype, formulation, adherence, drug combinations, and ethnicity as multifactorial contributors to CV, each exerting variable influence magnitudes (45). In terms of our study, modest reductions in %CV for MMF and SRL within the intervention cohort were observed, though these decreases were not statistically significant. When interpreting these results, it is critical to consider that pharmacokinetic variability of MMF is influenced by differences in albumin, bilirubin, and hemoglobin concentrations; renal and hepatic function; enterohepatic recirculation; co-administration of CsA; comorbidities such as cystic fibrosis; body weight; concomitant medications; time post-transplantation; gender; race; and genetic polymorphisms in drug-metabolizing enzymes. However, pharmacist-led interventions did not fully mitigate all confounding factors contributing to %CV variability. Furthermore, as stated in the label information, high-fat meals increase total SRL exposure by 23%–35%. Notably, SRL administration occurred post-discharge (≥3 months post-transplantation), and dietary standardization (e.g., fat intake) was not rigorously enforced in either study phase. Dietary factors likely contributed significantly to %CV fluctuations, potentially masking intervention effects. Nonetheless, pharmacist-led interventions partially mitigated modifiable confounders, resulting in a modest reduction in post-intervention %CV compared to pre-intervention.

To date, it has been demonstrated that the serum creatinine reduction rate determines the risk of rejection, displaying the dynamics of cystatin C and serum creatinine changes in the postoperative period (46, 47). Furthermore, urea nitrogen and microproteinuria have been proven to effectively predict graft function and clinical outcomes (48). In this study, we detected a downtrend of serum creatinine, cystatin C, and urea nitrogen in recipients with pharmacist intervention as early as one month after transplant, which persisted throughout the one-year time after the intervention of the pharmacists. A similar down trend of microproteinuria was also observed in the post-intervention group at 8 months after transplantation, indicating that the improvement of immunosuppressive management by pharmacists through various interventions could indirectly improve the outcome of grafts. This highlights the potential value of including a pharmacist in the transplantation team. There may be two reasons why microproteinuria increased significantly in the eighth month after kidney transplantation. Firstly, microalbuminuria may result from chronic rejection, reflecting glomerular basement membrane damage of a certain severity. Strictly speaking, C_min_ within the therapeutic target range and low %CV do not inherently preclude the occurrence of chronic rejection, as immunological and non-immunological mechanisms may independently drive graft injury. Secondly, the patient had a history of immune glomerular disease, which was closely related to graft loss and shortened survival time, and microproteinuria was one of the manifestations of postoperative glomerular disease activity.

The consensus report on therapeutic drug monitoring of mycophenolic acid in solid organ transplantation (19) states that the C_min_ (C_0_) method represents one of the monitoring approaches for mycophenolic acid (MPA). Its advantages include ease of implementation in clinical practice and requiring only a single sample. Its limitations are timing may not be accurate; timing may vary from the ideal 12-hour dose interval; and it does not exhibit a particularly strong association with the full area under the concentration-time curve (AUC). Due to these advantages, the C_min_ monitoring method was adopted in our center.

Acute rejection was not included in our results given that renal biopsy is not routinely performed for kidney transplant recipients at our center unless clinically indicated to confirm suspected acute rejection refractory to conventional immunosuppressive therapy (per KDIGO guideline recommendations). Furthermore, emergency procurement conditions precluded obtaining pre-transplant donor kidney biopsies for baseline analysis.

According to the KDIGO guidelines (Chapter 1: General Principles for the Management of Glomerular Disease), the assessment of kidney function (Section 1.2) explicitly demonstrates in Practice Point 1.2.3 that reliance on random “spot” urine collections for determining the protein-creatinine ratio is suboptimal due to inherent temporal variations in both urinary protein and serum creatinine excretion patterns. Due to the afore mentioned reasons, the urine protein-to-creatinine ratio was not measured in all patients, resulting in partial data unavailability. Consequently, this parameter was not adopted as an outcome measure in the evaluation.

## 5 Limitation

Some limitations have to be underlined. First, the study is retrospective in nature and subject to errors related to data capture, although all data was manually reviewed for accuracy and due to the single-center nature of this study, findings may not be precisely applicable to other transplant centers due to protocol and practice differences. Second, in this study, serum creatinine, urea, cystatin C, and microproteinuria were used as indicators to evaluate graft function, however, recipients were treated with combination regimens usually which could be a confounding factor for appropriately evaluating other graft functions. We adjusted for potential drug interactions but cannot rule out potential interactions related to the patients’ polypharmacy. Third, other factors may have contributed to the improved 1-year graft survival between the two groups, making it a little difficult to determine the pharmacist’s share of the impact in this observation. Fourth, to address genetic confounding, population pharmacokinetic modeling via nonlinear mixed-effects methods should be applied to derive genotype-specific parameters for tailored dose optimization. While our current study did not implement this strategy – employing literature-based genotype adjustments instead – future studies will integrate pharmacogenomic-guided modeling to enhance therapeutic precision.

## 6 Conclusion

Delegating the management of immunosuppressants to clinical pharmacists is a viable alternative to primary management that warrants further consideration and investigation at larger centers with greater patient populations and more pharmacists to assess its feasibility on a larger scale.

## Data Availability

The datasets presented in this article are not readily available because “only individuals named in the ethics application are authorised to access the data”. Requests to access the datasets should be directed to H.C. at chxgx008@126.com.

## References

[1] Vranian SC Jr. Covert KL Mardis CR McGillicuddy JW Chavin KD Dubay D. Assessment of risk factors for increased resource utilization in kidney transplantation. J Surg Res. (2018) 222:195–202.e2. doi: 10.1016/j.jss.2017.09.037, PMID: 29100587 PMC5742052

[2] Cohen EA McKimmy D Cerilli A Kulkarni S. A pharmacist-driven intervention designed to improve medication accuracy in the outpatient kidney transplant setting. Drug Healthc Patient Saf. (2020) 12:229–35. doi: 10.2147/DHPS.S264022, PMID: 33269008 PMC7701366

[3] Kuypers DRJ. From nonadherence to adherence. Transplantation. (2020) 104:1330–40. doi: 10.1097/TP.0000000000003112, PMID: 31929426

[4] Lieb M Hepp T Schiffer M Opgenoorth M Erim Y. Accuracy and concordance of measurement methods to assess non-adherence after renal transplantation - a prospective study. BMC Nephrol. (2020) 21:114. doi: 10.1186/s12882-020-01781-1, PMID: 32234021 PMC7110822

[5] Mohapatra A Valson AT Annapandian VM David VG Alexander S Jacob S. Post-transplant complications, patient, and graft survival in pediatric and adolescent kidney transplant recipients at a tropical tertiary care center across two immunosuppression eras. Pediatr Transpl. (2021) 25:e13973. doi: 10.1111/petr.13973, PMID: 33463876 PMC7615901

[6] Kostalova B Mala-Ladova K Kubena AA Horne R Dusilova Sulkova S Maly J. Changes in beliefs about post-transplant immunosuppressants over time and its relation to medication adherence and kidney graft dysfunction: a follow-up study. Patient Prefer Adherence. (2021) 15:2877–87. doi: 10.2147/PPA.S344878, PMID: 35002225 PMC8725840

[7] Chen T Wang Y Tian D Zhang J Xu Q Lv Q. Follow-up factors contribute to immunosuppressant adherence in kidney transplant recipients. Patient Prefer Adherence. (2022) 16:2811–9. doi: 10.2147/PPA.S383243, PMID: 36284546 PMC9588292

[8] Sanders-Pinheiro H Colugnati FAB Denhaerynck K Marsicano EO Medina JOP De Geest S. Multilevel correlates of immunosuppressive nonadherence in kidney transplant patients: the multicenter ADHERE BRAZIL study. Transplantation. (2021) 105:255–66. doi: 10.1097/TP.0000000000003214, PMID: 32150041

[9] Torres-Gutierrez M Burgos-Camacho V Caamano-Jaraba JP Lozano-Suarez N Garcia-Lopez A Giron-Luque F. Prevalence and modifiable factors for holistic non-adherence in renal transplant patients: a cross-sectional study. Patient Prefer Adherence. (2023) 17:2201–13. doi: 10.2147/PPA.S419324, PMID: 37701427 PMC10493132

[10] Yang H Li L Hu X Wang W Yang X Liu H. Impact of pharmacist-led post-transplant medication management for kidney transplant recipients: a retrospective pre- and post-intervention study. J Clin Pharm Ther. (2019) 44:603–10. doi: 10.1111/jcpt.12826, PMID: 30883843

[11] Lichvar AB Chandran MM Cohen EA Crowther BR Doligalski CT Condon Martinez AJ. The expanded role of the transplant pharmacist: a 10-year follow-up. Am J Transpl. (2023) 23:1375–87. doi: 10.1016/j.ajt.2023.04.032, PMID: 37146942 PMC11851232

[12] Rodriguez KE Chelewski RJ Peter ME Zuckerman AD Choi L DeClercq J. Integrating pharmacists into a kidney transplant clinic: developing and implementing a collaborative pharmacy practice agreement. J Am Pharm Assoc (2003). (2022) 62:349–56. doi: 10.1016/j.japh.2021.07.004, PMID: 34340924

[13] Taber DJ Fleming JN Su Z Mauldin P McGillicuddy JW Posadas A. Significant hospitalization cost savings to the payer with a pharmacist-led mobile health intervention to improve medication safety in kidney transplant recipients. Am J Transpl. (2021) 21:3428–35. doi: 10.1111/ajt.16737, PMID: 34197699

[14] Urzi Brancati V Scarpignato C Minutoli L Pallio G. Use of pharmacogenetics to optimize immunosuppressant therapy in kidney-transplanted patients. Biomedicines. (2022) 10:1798. doi: 10.3390/biomedicines10081798, PMID: 35892699 PMC9332547

[15] KDIGO. KDIGO clinical practice guideline for the care of kidney transplant recipients. Am J Transpl. (2009) 9 Suppl 3:S1–155. 10.1111/j.1600-6143.2009.02834.x 19845597

[16] Jing Y Kong Y Hou X Liu H Fu Q Jiao Z. Population pharmacokinetic analysis and dosing guidelines for tacrolimus co-administration with Wuzhi capsule in Chinese renal transplant recipients. J Clin Pharm Ther. (2021) 46:1117–28. doi: 10.1111/jcpt.13407, PMID: 33768546

[17] Wiseman A Alhamad T Alloway RR Concepcion BP Cooper M Formica R. Use of LCP-tacrolimus (LCPT) in kidney transplantation: a Delphi consensus survey of expert clinicians. Ann Transpl. (2024) 29:e943498. doi: 10.12659/AOT.943498, PMID: 38526543 PMC10944009

[18] Nguyen TVA Nguyen HD Nguyen TLH Le VT Nguyen XK Tran VT. Higher tacrolimus trough levels and time in the therapeutic range are associated with the risk of acute rejection in the first month after renal transplantation. BMC Nephrol. (2023) 24:131. doi: 10.1186/s12882-023-03188-0, PMID: 37158838 PMC10169362

[19] Kuypers DR Le Meur Y Cantarovich M Tredger MJ Tett SE Cattaneo D. Consensus report on therapeutic drug monitoring of mycophenolic acid in solid organ transplantation. Clin J Am Soc Nephrol. (2010) 5:341–58. doi: 10.2215/CJN.07111009, PMID: 20056756

[20] Liu J Feng D Kan X Zheng M Zhang X Wang Z. Polymorphisms in the CYP3A5 gene significantly affect the pharmacokinetics of sirolimus after kidney transplantation. Pharmacogenomics. (2021) 22:903–12. doi: 10.2217/pgs-2021-0083, PMID: 34523354

[21] Lhermitte R Le Dare B Laval F Lemaitre F Troussier B Morin MP. A pharmacist-led intervention to improve kidney transplant recipient outcomes and identify patients at risk of highly variable trough tacrolimus levels: a cohort study. Eur J Hosp Pharm. (2024) 31:314–20. doi: 10.1136/ejhpharm-2022-003625, PMID: 36737230 PMC11265551

[22] Chambord J Chauveau B Djabarouti S Vignaud J Taton B Moreau K. Measurement of the immunosuppressant possession ratio by transplant clinical pharmacists captures a non-adherence associated with antibody-mediated rejection. Transpl Int. (2023) 36:11962. doi: 10.3389/ti.2023.11962, PMID: 38089004 PMC10713790

[23] Gawedzki P Collins J. Impact of the implementation of a pharmacist-driven immunosuppression drug monitoring protocol for hematopoietic stem cell transplant recipients. J Oncol Pharm Pract. (2021) 27:1907–13. doi: 10.1177/1078155220975088, PMID: 33250016

[24] Taber DJ Pilch NA McGillicuddy JW Mardis C Treiber F Fleming JN. Using informatics and mobile health to improve medication safety monitoring in kidney transplant recipients. Am J Health Syst Pharm. (2019) 76:1143–9. doi: 10.1093/ajhp/zxz115, PMID: 31361870

[25] Fleming JN Gebregziabher M Posadas A Su Z McGillicuddy JW Taber DJ. Impact of a pharmacist-led, mHealth-based intervention on tacrolimus trough variability in kidney transplant recipients: a report from the TRANSAFE Rx randomized controlled trial. Am J Health Syst Pharm. (2021) 78:1287–93. doi: 10.1093/ajhp/zxab157, PMID: 33821958 PMC8599187

[26] Chambord J Couzi L Merville P Moreau K Xuereb F Djabarouti S. Benefit of a pharmacist-led intervention for medication management of renal transplant patients: a controlled before-and-after study. Ther Adv Chronic Dis. (2021) 12:20406223211005275. doi: 10.1177/20406223211005275, PMID: 33868624 PMC8024450

[27] Shigematsu T Suetsugu K Yamamoto N Tsuchiya Y Masuda S. Comparison of 4 commercial immunoassays used in measuring the concentration of tacrolimus in blood and their cross-reactivity to its metabolites. Ther Drug Monit. (2020) 42:400–6. doi: 10.1097/FTD.0000000000000696, PMID: 31568181

[28] Udomkarnjananun S Eiamsitrakoon T de Winter BCM van Gelder T Hesselink DA. Should we abandon therapeutic drug monitoring of tacrolimus in whole blood and move to intracellular concentration measurements? Br J Clin Pharmacol. (2025) 91:1530–41., PMID: 37897055 10.1111/bcp.15946

[29] Brunet M van Gelder T Åsberg A Haufroid V Hesselink DA Langman L. Therapeutic drug monitoring of tacrolimus-personalized therapy: second consensus report. Ther Drug Monit. (2019) 41:261–307. doi: 10.1097/FTD.0000000000000640, PMID: 31045868

[30] Ferreira PCL Thiesen FV Pereira AG Zimmer AR Froehlich PE. A short overview on mycophenolic acid pharmacology and pharmacokinetics. Clin Transpl. (2020) 34:e13997. doi: 10.1111/ctr.13997, PMID: 32484985

[31] Lloberas N Grinyo JM Colom H Vidal-Alabro A Fontova P Rigo-Bonnin R. A prospective controlled, randomized clinical trial of kidney transplant recipients developed personalized tacrolimus dosing using model-based Bayesian prediction. Kidney Int. (2023) 104:840–50. doi: 10.1016/j.kint.2023.06.021, PMID: 37391040

[32] Brunet M Pastor-Anglada M. Insights into the pharmacogenetics of tacrolimus pharmacokinetics and pharmacodynamics. Pharmaceutics. (2022) 14:1755. doi: 10.3390/pharmaceutics14091755, PMID: 36145503 PMC9503558

[33] Tecen-Yucel K Bayraktar-Ekincioglu A Yildirim T Yilmaz SR Demirkan K Erdem Y. Assessment of clinically relevant drug interactions by online programs in renal transplant recipients. J Manage Care Spec Pharm. (2020) 26:1291–6. doi: 10.18553/jmcp.2020.26.10.1291, PMID: 32996393 PMC10390948

[34] Chen H Qiu X Wang J Wei H. Pharmacists’ role in multidisciplinary diagnosis and treatment in adverse reactions: a case report of interferon alfa-2b induced severe lupus. Med (Baltimore). (2022) 101:e31997. doi: 10.1097/MD.0000000000031997, PMID: 36550841 PMC9771234

[35] Alsheikh R Johnson K Dauenhauer A Kadambi P. Impact of transplant pharmacists on length of stay and 30-day hospital readmission rate: a single-centre retrospective cohort study. Eur J Hosp Pharm. (2021) 28:e146–50. doi: 10.1136/ejhpharm-2020-002421, PMID: 33380430 PMC8640391

[36] Denson A Taylor MC Arnall J. Considerations for the role of the pharmacist in managing patients on eculizumab for hematopoietic stem cell transplantation-related thrombotic microangiopathy. Ann Pharmacother. (2023) 57:622–4. doi: 10.1177/10600280221123089, PMID: 36062453

[37] Pourrat X Berthy E Dupuis A Barbier L Buchler M Guillon LG. Implementing a personalized pharmaceutical plan in kidney or liver transplant patients: study protocol for a stepped-wedge cluster randomized trial (GRePH). Trials. (2021) 22:782. doi: 10.1186/s13063-021-05749-w, PMID: 34749777 PMC8573912

[38] Duwez M Chanoine S Lepelley M Vo TH Pluchart H Mazet R. Clinical evaluation of pharmacists’ interventions on multidisciplinary lung transplant outpatients’ management: results of a 7-year observational study. BMJ Open. (2020) 10:e041563. doi: 10.1136/bmjopen-2020-041563, PMID: 33247028 PMC7703423

[39] Kostalova B Mala-Ladova K Sulkova SD Denhaerynck K De Geest S Maly J. Comparison of different methods to assess tacrolimus concentration intra-patient variability as potential marker of medication non-adherence. Front Pharmacol. (2022) 13:973564. doi: 10.3389/fphar.2022.973564, PMID: 36313323 PMC9609782

[40] McGillicuddy JW Chandler JL Sox LR Taber DJ. Exploratory analysis of the impact of an mHealth medication adherence intervention on tacrolimus trough concentration variability: *post hoc* results of a randomized controlled trial. Ann Pharmacother. (2020) 54:1185–93. doi: 10.1177/1060028020931806, PMID: 32506922 PMC7814696

[41] Ko H Kim HK Chung C Han A Min SK Ha J. Association between medication adherence and intrapatient variability in tacrolimus concentration among stable kidney transplant recipients. Sci Rep. (2021) 11:5397. doi: 10.1038/s41598-021-84868-5, PMID: 33686160 PMC7940492

[42] Eaton CK Pruette CS Riekert KA Yousefzadeh N Pai AL Amaral S. Tacrolimus IPV group membership does not consistently track with electronically monitored or self-reported adherence in adolescent and young adult kidney transplant recipients. Transplant Direct. (2025) 11:e1806. doi: 10.1097/TXD.0000000000001806, PMID: 40371052 PMC12074067

[43] Vaisbourd Y Dahhou M Zhang X Sapir-Pichhadze R Cardinal H Johnston O. Differences in medication adherence by sex and organ type among adolescent and young adult solid organ transplant recipients. Pediatr Transpl. (2023) 27:e14446. doi: 10.1111/petr.14446, PMID: 36478059

[44] Kopfman M Brokhof M Patel S Fu D Olaitan O. High intrapatient tacrolimus variability and increased cell-free DNA in kidney transplant recipients. Prog Transpl. (2024) 34:204–10. doi: 10.1177/15269248241288559, PMID: 39376164

[45] Chen H Liu S Yu L Hou X Zhao R. Factors and interventions affecting tacrolimus intrapatient variability: a systematic review and meta-analysis. Transplant Rev (Orlando). (2024) 38:100878. doi: 10.1016/j.trre.2024.100878, PMID: 39260119

[46] Schold JD Nordyke RJ Wu Z Corvino F Wang W Mohan S. Clinical events and renal function in the first year predict long-term kidney transplant survival. Kidney360. (2022) 3:714–27. doi: 10.34067/KID.0007342021, PMID: 35721618 PMC9136886

[47] Garg N Poggio ED Mandelbrot D. The evaluation of kidney function in living kidney donor candidates. Kidney360. (2021) 2:1523–30. doi: 10.34067/KID.0003052021, PMID: 35373109 PMC8786144

[48] Mezzolla V Pontrelli P Fiorentino M Stasi A Franzin R Rascio F. Emerging biomarkers of delayed graft function in kidney transplantation. Transplant Rev (Orlando). (2021) 35:100629. doi: 10.1016/j.trre.2021.100629, PMID: 34118742

